# Association of serum uric acid to lymphocyte ratio, a novel inflammatory biomarker, with risk of stroke: A prospective cohort study

**DOI:** 10.1111/cns.14094

**Published:** 2023-01-17

**Authors:** Xue Tian, Penglian Wang, Shuohua Chen, Yijun Zhang, Xiaoli Zhang, Qin Xu, Yanxia Luo, Shouling Wu, Anxin Wang

**Affiliations:** ^1^ Department of Epidemiology and Health Statistics School of Public Health, Capital Medical University Beijing China; ^2^ Beijing Municipal Key Laboratory of Clinical Epidemiology Beijing China; ^3^ Department of Neurology, Beijing Tiantan Hospital Capital Medical University Beijing China; ^4^ China National Clinical Research Center for Neurological Diseases, Beijing Tiantan Hospital Capital Medical University Beijing China; ^5^ Department of Cardiology, Kailuan Hospital North China University of Science and Technology Tangshan China

**Keywords:** hemorrhagic stroke, mediation analysis, prospective cohort, serum uric acid to lymphocyte ratio, stroke

## Abstract

**Main Problem:**

Inflammation plays an important role in the pathological progress associated with stroke. Serum uric acid (SUA) to lymphocyte ratio (ULR), a novel inflammatory biomarker, has been considered as a better risk stratification tool of adverse outcomes than SUA or lymphocyte alone. This study aimed to investigate whether ULR produced more predictive value for stroke and explore the potential mediators of the associations.

**Methods:**

This study enrolled 93,023 Chinese participants without stroke and myocardial infarction at baseline. Cox proportional hazard models were used to analyze the associations of ULR with stroke and subtypes. Mediation analyses were conducted to explore potential mediators of the associations.

**Results:**

During a median follow‐up of 13.00 years, 6081 cases of incident stroke occurred, including 5048 cases of ischemic stroke (IS) and 900 cases of hemorrhagic stroke (HS). After adjustment for confounders, the Q4 group was associated with a higher risk of HS (HR, 1.25; 95% CI, 1.03–1.50), but not with total stroke (HR, 1.07; 95% CI, 1.03–1.13) or IS (HR, 1.04; 95% CI, 0.97–1.12). No significant associations were found between SUA or lymphocyte and any stroke. ULR outperformed SUA or lymphocytes alone in predicting stroke. Additionally, the significant association between ULR and HS was partially mediated by systolic blood pressure (20.32%), diastolic blood pressure (11.18%) and estimated glomerular filtration rate (9.19%).

**Conclusions:**

ULR was significantly associated with the risk of HS, but not with IS. Systolic blood pressure, diastolic blood pressure and estimated glomerular filtration rate were potential mediators for the association.

## Graphical abstract

Inflammation plays an important role in the pathological progress associated with stroke. Serum uric acid (SUA) to lymphocyte ratio (ULR), a novel inflammatory biomarker, has been considered as a better risk stratification tool of adverse outcomes than SUA or lymphocyte alone. Based on the Kailuan study, we enrolled 93,023 participants to investigate whether ULR produced more predictive value for stroke and explore the potential mediators of the associations. We found ULR was significantly associated with the risk of HS, but not with IS. ULR outperformed SUA or lymphocytes alone in predicting stroke. Systolic blood pressure, diastolic blood pressure, and estimated glomerular filtration rate were potential mediators for the association.

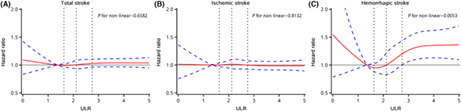

## 1 INTRODUCTION

Stroke is a disabling sequel of atherosclerosis with high morbidity and mortality rates worldwide, especially in East Asian countries.^1^ Given the complexity of stroke, different mechanisms are thought to be involved in this pathophysiology. In fact, increasing evidence shows that inflammation is involved in all stages of stroke.^2^, ^3^, ^4^, ^5^ Inflammation‐induced neuronal death is one of the key factors in stroke pathology.^6^ Therefore, inflammation control is a new strategy for reducing the occurrence and damage from stroke.

Serum uric acid (SUA) has been reported to have both antioxidant and pro‐inflammatory properties.^7^, ^8^ Elevated SUA is linked to inflammation and has been showed to be associated with metabolic syndrome, carotid atherosclerosis, endothelia dysfunction, oxidative stress and inflammation, which have adverse effects on platelet adhesiveness and aggregation.^9^, ^10^ Systemic inflammation response is characterized by increased neutrophil counts and decreased lymphocyte counts in chronic diseases.^11^, ^12^ And the neutrophil–lymphocyte ratio was reported to be associated with the prognosis of acute ischemic stroke in both clinical study and meta‐analysis.^13^, ^14^ Decrease in lymphocyte count reflects an impairment of adaptive immune system and poor general health status, and immune system is contributed to all levels of stroke cascade.^15^ However, it should be stated that the role of SUA or lymphocyte counts in stroke and subtypes has long been under debate.^16^, ^17^ Currently, SUA to lymphocyte ratio (ULR), showing a joint effect of SUA and lymphocyte count, has been considered as a novel risk stratification tool to refine prognostic prediction for heart disease and cancer. Additionally, ULR exhibited a better predictive ability than SUA or lymphocyte alone in these diseases.^18^, ^19^ However, whether ULR could be used as a novel inflammatory biomarker to produce better predictive value for stroke and subtypes has not been investigated up to now.

Therefore, based on a large community‐based population study, our present study sought to investigate the associations of ULR with stroke and its subtypes (ischemic stroke [IS] and hemorrhagic stroke [HS]), and to assess the potential mediators in the associations using mediation analysis.

## 2 METHODS

### 2.1 Study population

The data analyzed in this explorative study were retrieved from the Kailuan study, which is an ongoing prospective cohort study launched in Tangshan City, China. The detailed study designed and procedures have been described elsewhere.^20^, ^21^ Briefly, from June 2006 to October 2007, a total of 101,510 participants aged 18–98 years were enrolled from 11 affiliated hospitals of the Kailuan community. All the participants underwent questionnaire interviews, clinical examinations, and laboratory tests at enrollment, and were followed up every 2 years since baseline (2006). Participants meeting the following criteria were excluded^1^: had a history of stroke or MI (*n* = 3715)^2^; had missing data on SUA or lymphocytes at baseline (*n* = 4772). Finally, a total of 93,023 participants were enrolled in the current study (Figure S1). A comparison of baseline characteristics between excluded and included participants was presented in Table S1. The study was performed according to the guidelines of the Declaration of Helsinki and was approved by the Ethics Committee of Kailuan General Hospital and Beijing Tiantan Hospital. All participants provided written informed consent.

### 2.2 Assessment of SUA, lymphocytes, and ULR

Fasting blood samples were collected in the morning after an 8‐ to 12‐h overnight fast. The concentration of SUA was examined with a commercial kit (Ke Hua Biological Engineering Corporation, Shanghai, China) using an automatic biochemical analyzer (Hitachi 7600, Tokyo, Japan), according to the manufacturer's instructions. Lymphocytes were determined using a full blood count analyzer (Sysmex XT‐1800i, Sysmex Corporation). ULR was calculated as SUA (mg/dl)/lymphocyte count (×10^9^/L), as previously reported.^18^, ^19^

### 2.3 Assessment of outcomes

The primary outcome was the first occurrence of stroke (including IS, HS, and subarachnoid hemorrhage), either the first nonfatal stroke event, or stroke death without a preceding nonfatal event. The secondary outcomes were the individual endpoint of ischemic or HS. Subarachnoid hemorrhage events were not analyzed separately since the small sample size (*n* = 133). Participants were followed up via face‐to‐face interviews at every 2‐year interval, until 31 December 2019, or until the event of interest or death. Incident stroke events were ascertained by checking each year's discharge lists from the 11 local hospitals in Kailuan, medical records from medical insurance, or face‐to‐face interview on self‐reported history if disease. Vital status was collected from death certificates from the provincial vital statistic offices. The diagnosis of incident stroke was confirmed by medical review, using the World Health Organization criteria.^22^ Information on imaging diagnoses (including brain computerized tomography, magnetic resonance, or lumbar puncture) were collected to identify the type of incident stroke cases.

### 2.4 Assessment of covariates

Information on age, sex, education level, family income, smoking status, drinking status, physical activity, history of hypertension, diabetes, dyslipidemia, the use of antihypertensive, antidiabetic, or lipid‐lowering agents were collected using self‐reported questionnaires. Active physical activity was defined as ≥80 minutes of activity per week. Body mass index (BMI) was calculated as weight (kg)/ height (m)^2^. Systolic blood pressure (SBP) and diastolic blood pressure (DBP) were measured three times with the participants in the seated position using a mercury sphygmomanometer, and the average of three readings was used in the analyses.

Laboratory tests, including fasting blood glucose (FBG), total cholesterol (TC), triglycerides (TG), low‐density lipoprotein cholesterol (LDL‐C), high‐density lipoprotein cholesterol (HDL‐C), serum creatinine and high sensitivity C‐reactive protein (hs‐CRP) were assessed by an auto analyzer (Hitachi 747, Hitachi) at the central laboratory of Kailuan Hospital. Estimated glomerular filtration rate (eGFR) was calculated using the creatinine‐based Chronic Kidney Disease Epidemiological Collaboration (CKD‐EPI) equation.^23^

### 2.5 Statistical analysis

The baseline characteristics were described as the mean ± standard deviation for continuous variables, and frequencies with percentages for categorical variables. Participants were divided into four groups by quartiles of ULR. Person‐years was calculated from baseline to the first occurrence of stroke, mortality, or the end of the study (December 31, 2019), whichever came first. The incidence rate of stroke was calculated by dividing the number of incident cases by the total follow‐up duration (person‐years). A Cox proportional hazards model was used to estimate the associations of ULR with incident stroke and its subtypes. ULR was categorized in quartiles and was also modeled as a continuous variable in the analyses. Proportional hazards assumption was stratified by checking the Schoenfeld residual plots. Three models were built systematically, model 1 was unadjusted; model 2 was adjusted for age, sex, education, drinking status, smoking status, physical activity, BMI, SBP, DBP, FBG, TC, and HDL‐C; model 3 was further adjusted for history of hypertension, diabetes, dyslipidemia, medication on hypertension, diabetes, dyslipidemia, eGFR, and hs‐CRP. Restricted cubic spline with five knots (at the 5th, 25th, 50th, 75th, and 95th percentiles) was used to assess the shape of the associations between ULP and stroke.

A series of sensitivity analyses was performed to validate the robustness of our findings. First, the competing risk model was applied to with non‐stroke death being regarded as a competing risk event. Second, to explore the potential impact of reverse causality, we repeated the primary analysis using a 2‐year lagged period by excluding participants who developed stroke cases within the first 2 years of follow‐up. Third, we excluded population with an eGFR less than 30 ml/min/1.73 m^2^. Fourth, we excluded participants who used antihypertensive, antidiabetic, or lipid‐lowering agents to explore whether the association were confounded by medication use. Finally, we further excluded participants who had a history of cancer at baseline. Subgroup analyses stratified by age (<60 vs ≥60 years) and sex were performed, interaction of stratified variables with ULR was tested using likelihood test. The prognostic accuracy of ULR in predicting risk of stroke in terms of area under curve (AUC), sensitivity, and specificity was performed by using receiver operative characteristics curves, and the corresponding sensitivity and specificity were recorded with the largest Youden index. To explore whether ULR was better than SUA and lymphocyte count alone in predicting stroke, we compared the strength of the association of SUA, lymphocyte count, and ULR with the risk of stroke. Additionally, we used the C‐statistics, integrated discrimination improvement (IDI), and net reclassification index (NRI) to compare the incremental predictive value of SUA, lymphocyte count, and ULR beyond conventional risk factors.

Once the temporal relationships of ULR with stroke and its subtypes had been established, mediation analysis was performed to examine whether the associations between ULR (X) and stroke (Y) were mediated by other metabolic factors (M), using the method described by Valeri and VanderWeele.^24^, ^25^, ^26^ In general, four steps are involved in the mediation analysis^1^: demonstrating that the predictor is associated with the outcome (Model Y = *β*_Tol_ X, *β*_Tol_ = total effect)^2^; demonstrating that the predictor is associated with the mediator (Model M = *β*_1_ *X*, *β*_1_ = indirect effect 1)^3^; demonstrating which part of the outcome is explained by controlling for the predictor (Model Y = *β*_2_ M + *β*_Dir_ X, *β*_2_ = indirect effect 2, *β*_Dir_ = direct effect); and^4^ calculating the proportion of mediation: mediation effect (%) = (*β*_1_ × *β*_2_/*β*_Tol_) × 100%. We adjusted age, sex, education, income, smoking status, and drinking status in the mediation analysis, as it is necessary for mediation models in which baseline covariates are sufficient to control for exposure‐outcome, mediator‐outcome, and exposure‐mediator confounding.^27^, ^28^, ^29^

All statistical analyses were conducted using SAS version 9.4 software (SAS Institute, Cary, NC, USA). All reported *p*‐values were based on two‐sided tests of significance, and values of *p* < 0.05 were deemed statistically significant.

## 3 RESULTS

### 3.1 Baseline characteristics

A total of 93,023 participants were enrolled in the current study, 73,778 (79.31%) participants were men, and the mean age was 51.57 ± 12.55 years old. The baseline characteristics of participants according to quartiles of ULR was presented in Table 1. Compared with participants in the Q1 group, those with higher ULR tended to older, men, well‐educated, current smokers and drinkers, have a higher prevalence of hypertension, dyslipidemia, were more likely to take antihypertensive medication, have a higher level of BMI, SBP, DBP, TC, LDL‐C, hs‐CRP, but a lower prevalence of diabetes and a lower level of FBG, HDL‐C and eGFR.

**TABLE 1:** Baseline characteristics of the enrolled participants

Characteristics	Overall	Quartiles of ULR				*p* value
		Q1 (<1.63)	Q2 (1.63–2.11)	Q3 (2.12–2.74)	Q4 (≥2.75)	
Participants, *n* (%)	93,023	23,253	23,244	23,274	23,252	
Age, years	51.57 ± 12.55	49.51 ± 11.77	50.55 ± 12.07	51.65 ± 12.49	54.59 ± 13.24	<0.0001
Males, *n* (%)	73,778 (79.31)	16,378 (70.43)	17,789 (76.53)	19,087 (82.01)	20,524 (88.27)	<0.0001
High school or above, *n* (%)	12,665 (14.12)	2829 (12.55)	3125 (13.86)	3341 (14.83)	3370 (15.27)	<0.0001
Income ≥800 yuan/month, *n* (%)	6222 (6.93)	1293 (5.73)	1568 (6.95)	1693 (7.51)	1668 (7.55)	<0.0001
Current smoker, *n* (%)	30,424 (32.7)	6780 (29.16)	7673 (33.01)	8192 (35.20)	7776 (33.44)	<0.0001
Current alcohol, *n* (%)	33,110 (36.58)	6553 (28.97)	8028 (35.42)	9049 (39.82)	9477 (42.10)	<0.0001
Active physical activity, *n* (%)	13,499 (14.51)	2823 (12.14)	3231 (13.90)	3531 (15.17)	3914 (16.83)	<0.0001
Hypertension, *n* (%)	40,449 (43.48)	9773 (42.03)	9624 (41.40)	9965 (42.82)	11,085 (47.67)	<0.0001
Diabetes mellitus, *n* (%)	8404 (9.03)	2541 (10.93)	2097 (9.02)	1836 (7.89)	1929 (8.30)	<0.0001
Dyslipidemia, *n* (%)	32,061 (34.46)	7757 (33.36)	7830 (33.69)	8121 (34.89)	8352 (35.92)	<0.0001
Antihypertensive agents, *n* (%)	8766 (9.42)	1649 (7.09)	1965 (8.45)	2307 (9.91)	2845 (12.24)	<0.0001
Hypoglycemic agents, *n* (%)	1981 (2.13)	569 (2.45)	498 (2.14)	437 (1.88)	477 (2.05)	0.0003
Lipid‐lowering agents, *n* (%)	614 (0.66)	133 (0.57)	161 (0.69)	144 (0.62)	176 (0.76)	0.0699
Body mass index, kg/m^2^	25.03 ± 3.50	24.87 ± 3.52	24.99 ± 3.48	25.08 ± 3.47	25.17 ± 3.51	<0.0001
Systolic blood pressure, mmHg	130.81 ± 20.86	129.74 ± 20.60	129.88 ± 20.48	130.71 ± 20.84	132.92 ± 21.34	<0.0001
Diastolic blood pressure, mmHg	83.48 ± 11.76	83.01 ± 11.56	83.11 ± 11.62	83.50 ± 11.78	84.30 ± 12.03	<0.0001
Fasting blood glucose, mmol/L	5.46 ± 1.68	5.55 ± 1.92	5.45 ± 1.68	5.40 ± 1.53	5.43 ± 1.54	<0.0001
Total cholesterol, mmol/L	4.94 ± 1.15	4.89 ± 1.21	4.94 ± 1.13	4.96 ± 1.13	4.97 ± 1.11	<0.0001
Triglyceride, mmol/L	1.68 ± 1.38	1.66 ± 1.33	1.65 ± 1.34	1.67 ± 1.37	1.72 ± 1.47	<0.0001
LDL cholesterol, mmol/L	2.34 ± 0.92	2.35 ± 0.94	2.33 ± 0.92	2.33 ± 0.90	2.33 ± 0.93	0.0040
HDL cholesterol, mmol/L	1.55 ± 0.41	1.57 ± 0.41	1.55 ± 0.40	1.55 ± 0.41	1.54 ± 0.41	<0.0001
eGFR, ml/min/1.73m^2^	81.81 ± 25.77	81.67 ± 26.69	82.01 ± 24.84	82.4 ± 24.56	81.16 ± 26.88	<0.0001
hs‐CRP, mg/L	2.40 ± 6.54	2.34 ± 7.01	2.27 ± 6.02	2.30 ± 6.26	2.71 ± 6.79	<0.0001
SUA, mg/dl	4.85 ± 1.41	3.74 ± 0.98	4.55 ± 1.00	5.15 ± 1.12	5.98 ± 1.43	<0.0001
lymphocyte count, *10^9^/L	2.37 ± 2.71	3.21 ± 5.25	2.43 ± 0.54	2.14 ± 0.47	1.71 ± 0.44	<0.0001

Abbreviations: eGFR, estimated glomerular filtration rate; HDL, high density lipoprotein; hs‐CRP, high‐sensitivity C‐reactive protein; LDL, low density lipoprotein; SUA, serum uric acid; ULR, uric acid to lymphocyte count ratio.

### 3.2 Associations of ULR with stroke and its subtypes

**FIGURE 1:**
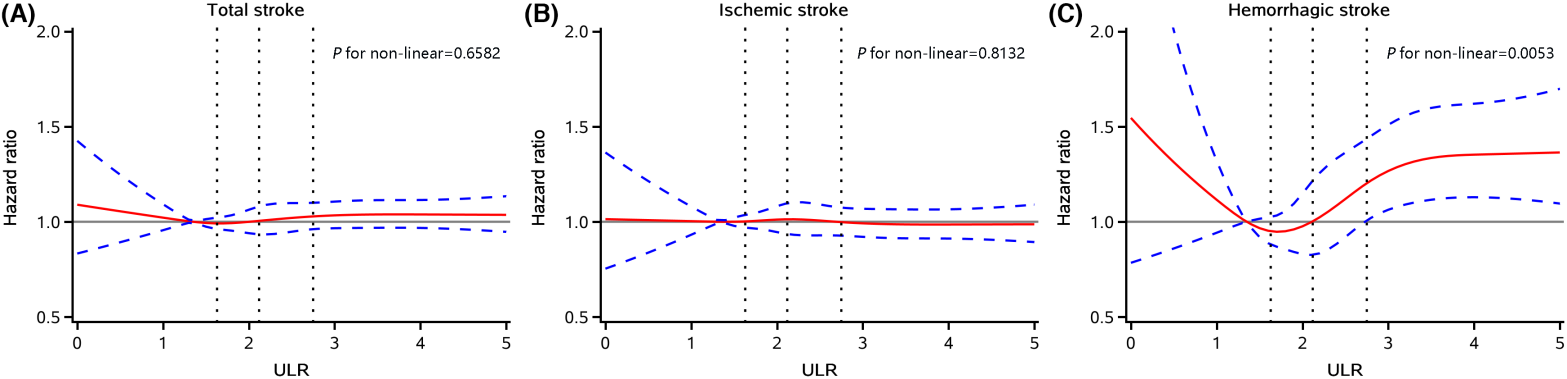
HRs and 95% CIs for ULR with risk of (A) total stroke, (B) ischemic stroke, and (C) hemorrhagic stroke by using restricted cubic spline regression. The red line represented HR and blue lines represented 95% CI. Adjusted for age, sex, education, drinking status, smoking status, physical activity, body mass index, systolic blood pressure, diastolic blood pressure, fasting blood glucose, total cholesterol, high density lipoprotein cholesterol, history of hypertension, diabetes, dyslipidemia, medication on hypertension, diabetes, dyslipidemia, estimated glomerular filtration rate, and high‐sensitivity C‐reactive protein. CI, confidence interval; HR, hazard ratio; ULR, serum uric acid to lymphocyte count ratio

During a median follow‐up of 13.00 (interquartile range, 12.61–13.18; range from 0.16–14.93 years) years, a total of 6081 stroke cases (6.54%) occurred, including 5048 cases IS and 900 cases of HS. The incidence per 1000 person‐years of total, ischemic, and HS increased substantially with increasing ULR quartiles, ranging from 4.76, 3.97, and 0.66 in the Q1 group to 6.38, 5.13 and 1.05 in the Q4 group, respectively (Table 2). After adjusted for potential variables, participants in the Q4 group remained having a 25% higher risk of HS (adjusted HR, 1.25; 95% CI, 1.03–1.50, *p* for trend = 0.0050), compared with those in the Q1 group. Nevertheless, the association of ULR with total stroke (HR, 1.04; 95% CI, 0.97–1.12; *p* for trend = 0.3798) and IS (HR, 1.04; 95% CI, 0.96–1.12; *p* for trend = 0.7358) attenuated to an insignificant level. Multivariable adjusted spline regression models showed that there was a non‐linear association between ULR and HS (*p* for non‐linear = 0.0053), but not with total stroke and IS (Figure 1).

**TABLE 2:** Association between quartiles of ULR with the risk of stroke

Outcomes	Quartiles of ULR				Per 1 unit increase	*p* for trend
	Q1 (<1.63)	Q2 (1.63–2.11)	Q3 (2.12–2.74)	Q4 (≥2.75)		
Total stroke						
Cases, *n* (%)	1359 (5.84)	1385 (5.96)	1587 (6.82)	1750 (7.53)		
Incidence rate^a^	4.76 (4.51–5.02)	4.85 (4.60–5.11)	5.62 (5.35–5.90)	6.38 (6.09–6.69)		
Model 1	Reference	1.02 (0.95–1.10)	1.18 (1.10–1.27)	1.35 (1.26–1.45)	1.09 (1.07–1.11)	<0.0001
Model 2	Reference	0.96 (0.89–1.03)	1.01 (0.94–1.08)	1.04 (0.97–1.12)	1.01 (0.99–1.03)	0.3890
Model 3	Reference	0.96 (0.89–1.03)	1.01 (0.94–1.08)	1.04 (0.97–1.12)	1.01 (0.99–1.03)	0.3798
Ischemic stroke						
Cases, *n* (%)	1138 (4.89)	1168 (5.02)	1326 (5.70)	1416 (6.09)		
Incidence rate^a^	3.97 (3.74–4.20)	4.07 (3.84–4.31)	4.67 (4.43–4.93)	5.13 (4.87–5.40)		
Model 1	Reference	1.03 (0.95–1.11)	1.18 (1.09–1.28)	1.30 (1.21–1.41)	1.08 (1.06–1.10)	<0.0001
Model 2	Reference	0.96 (0.89–1.04)	0.96 (0.89–1.04)	1.03 (0.95–1.12)	0.99 (0.97–1.02)	0.6843
Model 3	Reference	0.96 (0.89–1.05)	0.96 (0.89–1.04)	1.04 (0.96–1.12)	0.99 (0.97–1.02)	0.7358
Hemorrhagic stroke						
Cases, *n* (%)	191 (0.82)	185 (0.80)	229 (0.98)	295 (1.27)		
Incidence rate^a^	0.66 (0.57–0.76)	0.64 (0.55–0.73)	0.79 (0.70–0.90)	1.05 (0.94–1.18)		
Model 1	Reference	0.97 (0.79–1.19)	1.21 (1.00–1.46)	1.59 (1.33–1.91)	1.12 (1.08–1.15)	<0.0001
Model 2	Reference	0.94 (0.76–1.15)	1.10 (0.91–1.34)	1.26 (1.04–1.51)	1.08 (1.03–1.13)	0.0038
Model 3	Reference	0.94 (0.76–1.15)	1.10 (0.90–1.33)	1.25 (1.03–1.50)	1.07 (1.03–1.13)	0.0050

Abbreviation: ULR, uric acid to lymphocyte count ratio.

*Note*: Model 1: unadjusted.

Model 2: adjusted for age, sex, education, drinking status, smoking status, physical activity, body mass index, systolic blood pressure, diastolic blood pressure, fasting blood glucose, total cholesterol, and high density lipoprotein cholesterol.

Model 3: further adjusted for history of hypertension, diabetes, dyslipidemia, medication on hypertension, diabetes, dyslipidemia, estimated glomerular filtration rate, and high‐sensitivity C‐reactive protein.

^a^ Incidence rate per 1000 person‐years.

**FIGURE 2:**
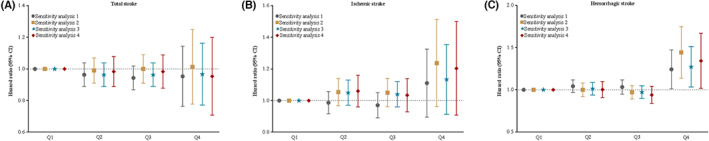
Sensitivity analysis for the association of ULR with stroke and subtypes. Sensitivity 1: Taking non‐stroke related death as competing risk event rather than censoring. Sensitivity 2: Excluded person time and incident stroke cases from the first 2 years of follow‐up (*n* = 91,788 for analysis). Sensitivity 3: Excluded participants with estimated glomerular filtration rate < 30 ml/min/1.73m^2^ (*n* = 92,497 for analysis). Sensitivity 4: Excluded participants with medication on hypertension, diabetes, dyslipidemia (*n* = 70,493 for analysis). Sensitivity 5: Excluded participants with a history of cancer (*n* = 92,709 for analysis). Adjusted for age, sex, education, drinking status, smoking status, physical activity, body mass index, systolic blood pressure, diastolic blood pressure, fasting blood glucose, total cholesterol, high density lipoprotein cholesterol, history of hypertension, diabetes, dyslipidemia, medication on hypertension, diabetes, dyslipidemia, estimated glomerular filtration rate, and high‐sensitivity C‐reactive protein

The sensitivity analyses using competing risk model, excluding participants who developed stroke cases within the first 2 years of follow‐up (*n* = 1245), excluding those with eGFR less than 30 ml/min/1.73 m^2^ (*n* = 526), excluding those who used antihypertensive, antidiabetic, or lipid‐lowering agents (*n* = 22,530), excluded those with a history of cancer (*n* = 314) all generated similar findings with the primary analysis (Figure 2, Table S2). Subgroup analyses stratified by age and sex showed that the associations of ULR with stroke were similar to the full cohort and consistent across different subgroups, *p* values for tests of two‐way interaction effects of ULR by age and sex on total, ischemic, and HS were all >0.05, indicating no significant effect modification of the association between ULR and stroke (Table S3).

### 3.3 Comparisons of the associations between SUA, lymphocytes, ULR, and stroke

We compared the associations of SUA, lymphocytes, and ULR with the risk of stroke, the results showed that the risk of total stroke, IS, and HS was not significantly increased with increasing SUA (*p* for trend = 0.2528, 0.4683, and 0.2258, respectively) or lymphocytes (*p* for trend = 0.2641, 0.8542, and 0.1534, respectively), whereas the risk of HS was significantly increased with increasing ULR (*p* for trend = 0.0050) (Table S4).

The prognostic accuracy of ULR in predicting risk of stroke in terms of area AUC, sensitivity, and specificity is presented in Figure S2. We also compared the incremental predictive value of SUA, lymphocytes, and ULR beyond the conventional risk factors. When HS was the outcome of interest, the C‐statistics by the conventional model significantly improved with the addition of ULR (from 0.701 to 0.706, *p* = 0.0028), but not significantly improved with the addition of SUA (*p* = 0.1000) and lymphocytes (*p* = 0.2384). The discriminatory power and risk reclassification appeared to substantially better with the addition of ULR (IDI, 0.02%; 95% CI, 0.01%–0.04%; *p* = 0.0045; NRI, 14.80; 95% CI, 8.25–21.34; *p* < 0.0001), but not with the addition of either SUA (*p* = 0.1789 for IDI, and 0.3345 for NRI) or lymphocytes (*p* = 0.1651 for IDI, and 0.2564 for NRI). We also found that the C‐statistics (*p* = 0.0358), IDI (*p* = 0.0251), NRI (*p* = 0.0010) were significantly improved by the addition of ULR into the conventional model with SUA and lymphocyte count, indicating that the ULR had a higher predicting value than single SUA, lymphocyte count, and the combination of the two biomarkers (Table 3 and Figure S3). When total stroke or IS was the outcomes of interest, the addition of either SUA, lymphocytes, or ULR was not significantly improved the predictive value of conventional model (Table 3).

**TABLE 3:** Reclassification and discrimination statistics for ULR compared with SUA and lymphocyte count

Outcomes	C statistics		IDI		Category‐free NRI	
	Estimate (95% CI)	*p*	Estimate (95% CI), %	*p*	Estimate (95% CI), %	*p*
Total stroke						
Conventional model^a^	0.722 (0.716–0.728)		Reference		Reference	
Conventional model + SUA	0.722 (0.716–0.728)	0.5507	0.00 (0.00–0.00)	0.7953	1.29 (−3.89–1.30)	0.3292
Conventional model +LY	0.722 (0.716–0.728)	0.3978	0.00 (0.00–0.00)	0.6849	4.07 (−2.95–2.13)	0.7585
Conventional model +ULR	0.723 (0.717–0.728)	0.2875	0.01 (0.00–0.00)	0.7813	2.60 (0.04–5.17)	0.0494
Ischemic stroke						
Conventional model^a^	0.721 (0.715–0.727)					
Conventional model + SUA	0.721 (0.715–0.728)	0.7625	0.00 (0.00–0.00)	0.6537	2.44 (−0.38–5.28)	0.0908
Conventional model +LY	0.721 (0.715–0.728)	0.6967	0.00 (0.00–0.01)	0.5607	0.36 (−0.24–3.13)	0.8059
Conventional model +ULR	0.721 (0.715–0.728)	0.3708	0.00 (0.00–0.01)	0.4851	4.63 (1.83–7.43)	0.0014
Hemorrhagic stroke						
Conventional model^a^	0.701 (0.685–0.718)		Reference		Reference	
Conventional model + SUA	0.704 (0.688–0.721)	0.1000	0.01 (0.00–0.01)	0.1789	2.03 (−1.03–5.15)	0.3345
Conventional model +LY	0.703 (0.687–0.720)	0.2384	0.00 (0.00–0.02)	0.1651	2.29 (−0.42–18.74)	0.2564
Conventional model +ULR	0.706 (0.690–0.723)	0.0028	0.02 (0.01–0.04)	0.0045	14.80 (8.25–21.34)	<0.0001
Conventional model+SUA + LY	0.704 (0.688–0.722)		Reference		Reference	
Conventional model +SUA + LY + ULR	0.706 (0.691–0.723)	0.0358	0.01 (0.00–0.01)	0.0251	4.36 (1.80–6.93)	0.0010

Abbreviations: IDI, integrated discrimination improvement; LY, lymphocyte count; NRI, net reclassification index; SUA, serum uric acid; ULR, uric acid to lymphocyte count ratio.

^a^ Conventional model was adjusted for age; sex; education; income; smoking status; drinking status; physical activity, history of diabetes and dyslipidemia; medication on hypertension, diabetes, and dyslipidemia; body mass index; fasting blood glucose; systolic blood pressure; diastolic blood pressure; total cholesterol; high density lipoprotein cholesterol; estimated glomerular filtration rate; and high‐sensitivity C‐reactive protein.

### 3.4 Mediation analysis between ULR and HS

**FIGURE 3:**
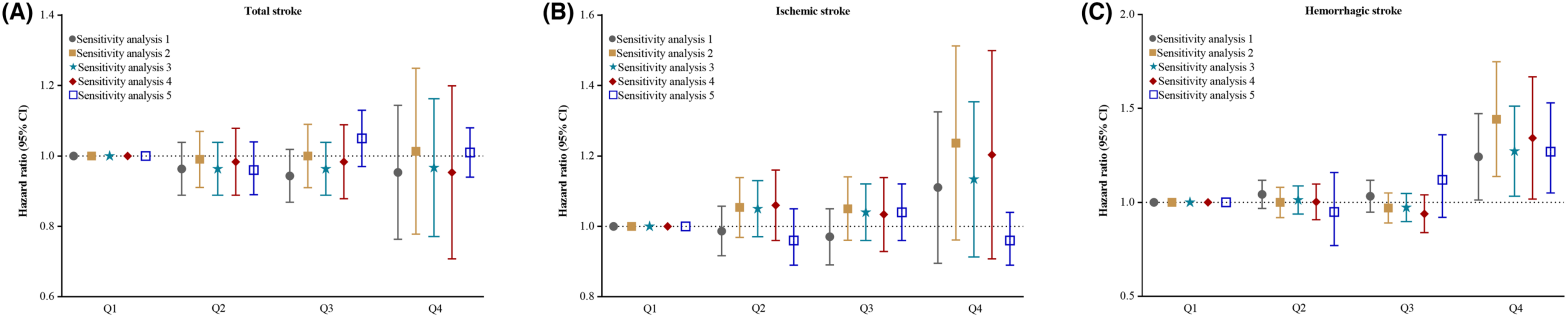
Mediation analyses of the association of ULR with stroke and subtypes. (A) Contribution of SBP; (B) Contribution of DBP; (C) Contribution of eGFR. Adjusted for age, sex, education, drinking status, smoking status, physical activity, body mass index, systolic blood pressure, diastolic blood pressure, fasting blood glucose, total cholesterol, high density lipoprotein cholesterol, history of hypertension, diabetes, dyslipidemia, medication on hypertension, diabetes, dyslipidemia, estimated glomerular filtration rate, and high‐sensitivity C‐reactive protein. ULR, serum uric acid to lymphocyte count ratio; SBP, systolic blood pressure; DBP, diastolic blood pressure; eGFR, estimated glomerular filtration rate. *p < 0.05; **p < 0.01

Since ULR was significantly associated with the risk of HS, mediation analysis was then used to assess the potential mechanisms. We tested BMI, SBP, DBP, FBG, TC, TG, LDL‐C, HDL‐C, eGFR, and hs‐CRP as potential mediators of the association between ULR and HS. The results showed that the total effect of ULR on HS was 0.0019 (95% CI, 0.0013–0.0026; *p* = 0.0010). The association between ULR and HS was partially mediated by SBP (*β*_indir_ = 0.0004, mediation proportion [MP] = 20.32%), DBP (*β*_indir_ = 0.0002, MP = 11.18%), and eGFR (*β*_indir_ = 0.0002, MP = 9.19%) (Figure 3). The remaining factors: BMI, FBG, TC, TG, LDL‐C, HDL‐C, and hs‐CRP did not play significant mediating roles in the association (Table S5).

## 4 DISCUSSION

In our present study, we prospectively investigated the association between ULR, a novel biomarker of inflammation and the risk of incident stroke. The major findings are listed as follows^1^: ULR was positively associated with the risk of HS, but not with either total stroke or IS, after adjustment for confounding risk factors^2^; ULR was a better predictor of HS than SUA or lymphocytes alone, the addition of ULR to the conventional model significantly promoted the ability of risk stratification^3^; the association between ULR and HS was partially mediated by SBP, DBP, and eGFR.

The immune‐biomarkers of stroke have drawn a lot of attention recently, such as lymphocyte,^30^ neutrophil,^31^ leukocyte,^32^ monocytes,^33^ T cell,^34^ and et al. Current literature on ULR is limited and restricted in patients after surgery. ULR was first proposed in the study of Wei et al., a retrospective study conducted in consecutive patients with rheumatic heart disease undergoing valve replacement surgery. The study with 949 elderly patients demonstrated that ULR, combining the effect of SUA and lymphocyte count, produced more prognostic value in elderly patients than SUA and lymphocyte count. Similarly, another study was carried out based on a prospectively‐maintained database and included 335 patients after video‐assisted thoracoscopic surgery lobectomy for early‐stage non‐small‐cell lung cancer, showing that elevated ULR could independently predict both unfavorable overall survival and disease‐free survival. The better predictive role of ULR was extended for primary prevention of stroke in our current study. Our results demonstrated that the risk of HS was significantly associated with a higher level of ULR, even adjustment for other potential confounders, but not with SUA or lymphocyte count. Furthermore, the addition of ULR into the conventional model yielded an incremental predictive value. Our findings suggested that application of ULR (a novel biomarker) could improve risk stratification ability in the primary prevention of HS.

We hypothesized that the biological reasons underlying the better predictive value of this ULR tool may be elucidated by a combination of the following three plausible mechanisms. First, SUA had both pro‐oxidant and antioxidant capabilities.^35^ Experimental studies have shown that increased SUA as a pro‐oxidant is associated with endothelial dysfunction, increased oxidative stress, elevated plasma renin activity and systemic inflammation mediators, which may contribute to the development of HS.^36^, ^37^ Population study found that elevated serum uric acid increases the risk of stroke recurrence.^38^ On the other hand, SUA is also an abundant natural antioxidant capable of reducing cellular oxidation, a major cause of neurodegenerative disease.^39^ The double‐edged properties may reduce the impact of SUA on the risk of HS, leading to the inconsistent association between SUA and HS.

Second, HS occurs when a blood vessel with the brain ruptures and releases blood.^40^ A systemic inflammation response, frequently accompanied with a sharp decline in peripheral lymphocyte count, has been proposed to be involve in the weakening of vessel wall and lead to vessel rupture, thus may increase the risk of HS.^41^ One study have found that the white blood cell count was significantly associated with deep intracerebral hemorrhage compared with small artery occlusion group.^41^ On the other hand, it is stated that lymphocyte count can mediate the inflammatory response, where it may increase the level of anti‐inflammatory cytokines and suppress the production of pro‐inflammatory cytokines.^42^ These may contribute to the controversial role of lymphocyte counts in the development of HS.

Finally, ULR, a combination of SUA and lymphocyte count, is elevated with the increase in SUA or the decrease in lymphocyte count, both of which were associated with a higher level of inflammation. An elevated SUA level has also been proved to show a strong relationship with a high circulating level of various pro‐inflammatory mediators, such as hs‐CRP.^43^ An inflammatory stress may also be generated when SUA enters cells due to the impact of intracellular SUA on the generation of reactive oxygen species to the reactive nitrogen species and the Cox‐2 activation.^44^ Decreased lymphocyte count implies a decline in immune regulation and an increase in stress.^45^ Taken together, the above facts strongly supported the premise, suggesting that a higher ULR may serve as a better risk stratification tool for HS.

Another striking finding with the present study is that ULR, as a novel biomarker, is significantly associated with the risk of HS, but not the risk of total stroke and IS. To further explore the potential mechanisms underlying the different associations, mediation analysis was performed, and the results showed that the association between ULR and HS was partially mediated by SBP, DBP, and eGFR, which can explain the different associations. Previous studies demonstrated that IS and HS have distinct risk profiles, hypertension and a level of low eGFR have been reported to be more strongly linked to HS than IS.^46^, ^47^ The main causes for the stronger correlation between hypertension and HS included micro‐aneurysm formed in cerebral arteriole at basal ganglion, weakened structure of external membrane and middle‐layer membrane of cerebral arterial wall, spasm of cerebral arteriole induced and fibrinoid necrosis of cerebral arteriole, high pulsating blood flow into the ruptured arteries in the brain can cause cerebral microvascular damage, and further lead to the occurrence of hemorrhage. ^48^, ^49^ Like the mechanisms of hypertension, low eGFR may induce platelet dysfunction leading to prolonged bleeding time and increasing risk of cerebral hemorrhages, or be correlated with cerebral small vessel disease, the mechanism behind most brain hemorrhages.^46^ Elevated ULR was associated with a higher level of SBP, DBP, and a lower level of eGFR, these factors contributed more to the pathophysiology of HS than IS.

Our study has several strengths. First, we investigated a novel biomarker, ULR with the risk of stroke, and we compared the incremental predictive value of SUA, lymphocyte, and ULR to. Furthermore, we quantified the contribution of other risk factors in the pathways between ULR and stroke to explore the potential mechanisms. However, several limitations should also be noted. First, some information was not available in the current study, such as urate‐lowering agents and reperfusion therapy, which could be considered in future investigations. Second, we separately assessed the mediating effects of an indicator of obesity, blood pressure, blood lipids, blood glucose, and an indicator of inflammation on the associations of ULR with stroke. However, because of the complexity associated with the numerous permutations of mediators, it may not be feasible to mutually consider the combined mediating and interactive effects of these mediators. Third, our study recruited much lower number of women than men. However, subgroup analysis stratified by sex showed that the associations were consistent across women and men. Fourth, selection bias may exist because participants with missing data on SUA or lymphocyte count were excluded. Finally, the study was conducted among Chinese population, thus the findings could not be generalized to other ethnicities. However, the constitution of the population was complex by consisting of individuals from all levels of society and across various occupations. Study of such a geographically confined and controlled population greatly reduces the residual confounding factors because of diverse socioeconomic factors and lifestyle patterns.

## 5 CONCLUSIONS

In conclusion, this explorative study found that ULR, serving as a novel inflammatory biomarker, was significantly associated with the risk of HS in Chinese adults, indicating that ULR can be employed as a simplified, effective, and routine risk stratification tool to provide readily objective information for the risk of HS. Additionally, the association between ULR and HS was partially mediated by SBP, DBP, and eGFR. These findings emphasized the important roles of ULR and metabolic factors as conjunctive intervention targets in the prevention of HS.

## AUTHOR CONTRIBUTIONS

YL, SW, and AW contributed to the conception and design of the study; XT contributed to manuscript drafting; XT, SC, YZ, XZ, and QX contributed to the statistics analysis; PW contributed to the acquisition of data; all authors contributed to critical revisions of the manuscript.

## FUNDING INFORMATION

This work was supported by National Key Research and Development Program of China (2022YFC3600600, 2022YFC3600603, 2018YFC1312400, and 2018YFC1312402), Training Fund for Open Projects at Clinical Institutes and Departments of Capital Medical University (CCMU2022ZKYXZ009), Beijing Natural Science Foundation Haidian original innovation joint fund (L222123), and Beijing Municipal Administration of Hospitals Incubating Program (PX2020021). The funder has no role in study design, data collection, data analysis, manuscript preparation, and/or publication decisions.

## CONFLICT OF INTEREST

None declared.

## Supporting information


Data S1:

## Data Availability

Data are available to researchers upon request for purposes of reproducing the results or replicating the procedure by directly contacting the corresponding author.
