# Supplementary material for: Association of serum uric acid to lymphocyte ratio, a novel inflammatory biomarker, with risk of stroke: A prospective cohort study
Source: CNS Neurosci Ther. 2023 Jan 17;29(4):1168–77. doi: 10.1111/cns.14094 (PMC10018086; doi:10.1111/cns.14094)
Supplement: Supplementary file 1 — Data S1: [file CNS-29-1168-s001.docx]

**Supplemental Materials**

Table S1. Baseline characteristics of the excluded and included participants

| Characteristics | Excluded  (n=8487) | Included  (n=93,023) | *P* value |
| --- | --- | --- | --- |
| Age, years | 55.86±13.27 | 51.57±12.55 | <0.0001 |
| Males, n (%) | 7332 (86.39) | 73778 (79.31) | <0.0001 |
| High school or above, n (%) | 1350 (17.37) | 12665 (14.12) | <0.0001 |
| Income≥800RMB, n (%) | 548 (7.05) | 6222 (6.93) | 0.6945 |
| Current smoker, n (%) | 3374 (39.75) | 30421 (32.70) | <0.0001 |
| Current alcohol, n (%) | 3545 (45.42) | 33107 (36.57) | <0.0001 |
| Active physical activity, n (%) | 1782 (21.00) | 13499 (14.51) | <0.0001 |
| Hypertension, n (%) | 4208 (49.59) | 40447 (43.48) | <0.0001 |
| Diabetes mellitus, n (%) | 1086 (12.80) | 8403 (9.033) | <0.0001 |
| Dyslipidemia, n (%) | 4333 (51.06) | 32060 (34.46) | <0.0001 |
| Antihypertensive agents, n (%) | 2548 (30.03) | 8766 (9.42) | <0.0001 |
| Hypoglycemic agents, n (%) | 500 (5.89) | 1981 (2.13) | <0.0001 |
| Lipid-lowering agents, n (%) | 349 (4.11) | 614 (0.66) | <0.0001 |
| Body mass index, kg/m^2^ | 25.22±3.44 | 25.03±3.50 | <0.0001 |
| Systolic blood pressure, mmHg | 134.10±23.33 | 130.81±20.86 | <0.0001 |
| Diastolic blood pressure, mmHg | 83.60±12.10 | 83.48±11.76 | 0.3989 |
| Fasting blood glucose, mmol/L | 5.75±1.79 | 5.46±1.68 | <0.0001 |
| Total cholesterol, mmol/L | 5.10±1.14 | 4.94±1.15 | <0.0001 |
| Triglyceride, mmol/L | 1.69±1.29 | 1.68±1.38 | 0.3913 |
| LDL cholesterol, mmol/L | 2.49±0.81 | 2.34±0.92 | <0.0001 |
| HDL cholesterol, mmol/L | 1.46±0.37 | 1.55±0.41 | <0.0001 |
| eGFR, mL/min/1.73m^2^ | 83.32±23.96 | 81.81±25.77 | <0.0001 |
| hs-CRP, mg/L | 2.49±5.55 | 2.40±6.54 | 0.2918 |

Abbreviations: eGFR, estimated glomerular filtration rate; HDL, high density lipoprotein; hs-CRP, high-sensitivity C-reactive protein; LDL, low density lipoprotein;

Table S2. Sensitivity analysis for the associations of ULR with stroke

| Outcomes | Q1 | Q2 | Q3 | Q4 | *P* for trend |
| --- | --- | --- | --- | --- | --- |
| Competing risk model |  |  |  |  |  |
| Total stroke | Reference | 0.96(0.89-1.04) | 0.98(0.92-1.06) | 1.04(0.97-1.12) | 0.6851 |
| Ischemic stroke | Reference | 0.94(0.87-1.02) | 0.97(0.89-1.05) | 1.03(0.95-1.12) | 0.6422 |
| Hemorrhagic stroke | Reference | 0.94(0.77-1.15) | 1.10(0.90-1.33) | 1.23(1.02-1.48) | 0.0032 |
| 2-year lagged (n=91788) |  |  |  |  |  |
| Total stroke | Reference | 0.99(0.91-1.07) | 1.05(0.97-1.14) | 1.00(0.92-1.08) | 0.6367 |
| Ischemic stroke | Reference | 1.00(0.91-1.09) | 1.05(0.96-1.14) | 0.97(0.89-1.05) | 0.6846 |
| Hemorrhagic stroke | Reference | 0.99(0.79-1.26) | 1.22(0.97-1.52) | 1.42(1.15-1.76) | 0.0267 |
| Excluding eGFR<30 ml/min/1.73m^2^(n=92497) | | | | | |
| Total stroke | Reference | 0.96(0.89-1.04) | 1.05(0.97-1.13) | 1.01(0.94-1.09) | 0.3019 |
| Ischemic stroke | Reference | 0.96(0.89-1.04) | 1.04(0.96-1.12) | 0.97(0.90-1.05) | 0.8174 |
| Hemorrhagic stroke | Reference | 0.95(0.78-1.17) | 1.12(0.92-1.36) | 1.26(1.04-1.52) | 0.0041 |
| Excluding medication (n=70493) | | | | | |
| Total stroke | Reference | 0.98(0.89-1.08) | 1.06(0.96-1.16) | 1.00(0.91-1.10) | 0.6616 |
| Ischemic stroke | Reference | 0.98(0.88-1.09) | 1.03(0.93-1.14) | 0.94(0.84-1.04) | 0.3728 |
| Hemorrhagic stroke | Reference | 0.93(0.72-1.21) | 1.18(0.92-1.51) | 1.32(1.03-1.68) | 0.0062 |
| Excluding participants with a history of cancer (n=92709) | | | |  |  |
| Total stroke | Reference | 0.96(0.89-1.04) | 1.05(0.97-1.13) | 1.01(0.94-1.08) | 0.3859 |
| Ischemic stroke | Reference | 0.96(0.89-1.05) | 1.04(0.96-1.12) | 0.96(0.89-1.04) | 0.6802 |
| Hemorrhagic stroke | Reference | 0.95(0.77-1.16) | 1.12(0.92-1.36) | 1.27(1.05-1.53) | 0.0029 |

eGFR, estimated glomerular filtration rate; ULR, serum uric acid to lymphocyte count ratio.

Adjusted for age, sex, education, drinking status, smoking status, physical activity, body mass index, systolic blood pressure, diastolic blood pressure, fasting blood glucose, total cholesterol, high density lipoprotein cholesterol, history of hypertension, diabetes, dyslipidemia, medication on hypertension, diabetes, dyslipidemia, estimated glomerular filtration rate, and high-sensitivity C-reactive protein.

Table S3. Association of quartiles of serum uric acid, lymphocyte count, and ULR with risk of stroke

| Outcomes | Q1 | Q2 | Q3 | Q4 | *P* for trend |
| --- | --- | --- | --- | --- | --- |
|  | Serum uric acid | | | | |
| Total stroke | Reference | 0.97(0.90-1.05) | 0.99(0.92-1.07) | 1.04(0.96-1.12) | 0.2528 |
| Ischemic stroke | Reference | 0.97(0.90-1.06) | 0.99(0.91-1.08) | 1.02(0.94-1.11) | 0.4683 |
| Hemorrhagic stroke | Reference | 0.92(0.76-1.12) | 1.02(0.84-1.24) | 1.09(0.90-1.33) | 0.2258 |
|  | Lymphocyte count | | | | |
| Total stroke | Reference | 0.97(0.90-1.04) | 0.92(0.85-0.99) | 0.97(0.91-1.04) | 0.2641 |
| Ischemic stroke | Reference | 1.01(0.93-1.09) | 0.96(0.88-1.04) | 1.01(0.94-1.09) | 0.9542 |
| Hemorrhagic stroke | Reference | 0.83(0.70-1.09) | 0.78(0.60-1.04) | 0.80(0.67-1.06) | 0.1534 |
|  | ULR | | | | |
| Total stroke | Reference | 0.96(0.89-1.03) | 1.01(0.94-1.08) | 1.04(0.97-1.12) | 0.3798 |
| Ischemic stroke | Reference | 0.96(0.89-1.05) | 0.96(0.89-1.04) | 1.04(0.96-1.12) | 0.7358 |
| Hemorrhagic stroke | Reference | 0.94(0.76-1.15) | 1.10(0.90-1.33) | 1.25(1.03-1.50) | 0.0050 |

Abbreviations: ULR, uric acid to lymphocyte count ratio.

Adjusted for age, sex, education, drinking status, smoking status, physical activity, body mass index, systolic blood pressure, diastolic blood pressure, fasting blood glucose, total cholesterol, high density lipoprotein cholesterol, history of hypertension, diabetes, dyslipidemia, medication on hypertension, diabetes, dyslipidemia, estimated glomerular filtration rate, and high-sensitivity C-reactive protein.

Table S4. Age- and sex-specific association between high ULR and stroke

| Outcomes | Quartiles of ULR | | | | *P* for interaction |
| --- | --- | --- | --- | --- | --- |
|  | Q1 | Q2 | Q3 | Q4 |  |
| Age<60 years |  |  |  |  |  |
| Total stroke | Reference | 0.96(0.88-1.05) | 1.05(0.96-1.15) | 1.01(0.92-1.11) | 0.8350 |
| Ischemic stroke | Reference | 0.94(0.85-1.04) | 1.02(0.92-1.12) | 0.95(0.86-1.05) | 0.7666 |
| Hemorrhagic stroke | Reference | 1.05(0.82-1.34) | 1.22(0.96-1.55) | 1.36(1.07-1.73) | 0.3941 |
| Age≥60 years |  |  |  |  |  |
| Total stroke | Reference | 0.96(0.84-1.10) | 1.03(0.91-1.17) | 1.04(0.92-1.17) | 0.9812 |
| Ischemic stroke | Reference | 1.02(0.89-1.18) | 1.09(0.95-1.25) | 1.04(0.91-1.19) | 0.9571 |
| Hemorrhagic stroke | Reference | 0.75(0.53-1.07) | 0.90(0.65-1.25) | 1.08(0.80-1.46) | 0.6316 |
| Men |  |  |  |  |  |
| Total stroke | Reference | 0.97(0.89-1.05) | 1.05(0.97-1.13) | 1.02(0.94-1.10) |  |
| Ischemic stroke | Reference | 0.97(0.89-1.06) | 1.03(0.95-1.13) | 0.97(0.89-1.05) |  |
| Hemorrhagic stroke | Reference | 0.97(0.77-1.22) | 1.15(0.93-1.42) | 1.32(1.07-1.62) |  |
| Women |  |  |  |  |  |
| Total stroke | Reference | 0.93(0.76-1.12) | 1.02(0.84-1.25) | 0.95(0.76-1.20) |  |
| Ischemic stroke | Reference | 0.94(0.75-1.16) | 1.06(0.84-1.33) | 0.96(0.74-1.25) |  |
| Hemorrhagic stroke | Reference | 0.84(0.52-1.34) | 0.91(0.56-1.49) | 0.89(0.51-1.56) |  |

Abbreviations: ULR, uric acid to lymphocyte count ratio.

Adjusted for age, sex, education, drinking status, smoking status, physical activity, body mass index, systolic blood pressure, diastolic blood pressure, fasting blood glucose, total cholesterol, high density lipoprotein cholesterol, history of hypertension, diabetes, dyslipidemia, medication on hypertension, diabetes, dyslipidemia, estimated glomerular filtration rate, and high-sensitivity C-reactive protein.

Table S5. Direct and indirect effects of ULR on the risk of stroke and the proportion mediated by cardio-metabolic factors

| Potential mediators | Direct effect | |  | Indirect effect | |  | Proportion mediated (%) | *P* value |
| --- | --- | --- | --- | --- | --- | --- | --- | --- |
|  | β_dir,_ (95% CI) | *P* |  | β_indir_, (95% CI) | *P* |  |  |  |
| SBP | 0.0015(0.0008-0.0022) | <0.0001 |  | 0.0004(0.0003-0.0004) | <0.0001 |  | 20.32 | <0.0001 |
| DBP | 0.0017(0.0011-0.0023) | <0.0001 |  | 0.0002(0.0002-0.0003) | <0.0001 |  | 11.18 | <0.0001 |
| eGFR | 0.0017(0.0012-0.0025) | <0.0001 |  | 0.0002(0.0001-0.0003) | 0.0031 |  | 9.19 | 0.0028 |
| hs-CRP | 0.0018(0.0012-0.0025) | <0.0001 |  | 0.0001(0.000-0.0001) | 0.0026 |  | 1.82 | 0.0072 |
| BMI | 0.0019(0.0012-0.0025) | <0.0001 |  | 0.0000(0.0000-0.0001) | 0.0005 |  | 1.56 | 0.0027 |
| TG | 0.0019(0.0013-0.0026) | <0.0001 |  | 0.0000(-0.0001-0.0000) | 0.0816 |  | -- | -- |
| TC | 0.0019(0.0013-0.0025) | <0.0001 |  | 0.0000(-0.0001-0.0001) | 0.4138 |  | -- | -- |
| LDL-C | 0.0019(0.0013-0.0026) | <0.0001 |  | 0.0000(-0.0001-0.0001) | 0.0892 |  | -- | -- |
| HDL-C | 0.0019(0.0013-0.0026) | <0.0001 |  | -0.0001(-0.0001-0.0000) | 0.0771 |  | -- | -- |
| FBG | 0.0019(0.0013-0.0026) | <0.0001 |  | 0.0001(-0.0002-0.0000) | 0.0511 |  | -- | -- |

Abbreviations: BMI, body mass index; CI, confidence interval; CVD, cardiovascular disease; DBP, diabolic blood pressure; FBG, fasting blood glucose; TC, total cholesterol; TG, triglyceride; HDL-C, high density lipoprotein cholesterol; hs-CRP, high sensitivity C-reactive protein; LDL-C, low density lipoprotein cholesterol; SBP, systolic blood pressure.

Total effect was 0.0019(95% CI, 0.0013-0.0026; *P*=0.0010).

Adjusted for age, sex, education, income, smoking status, drinking status, and physical activity.


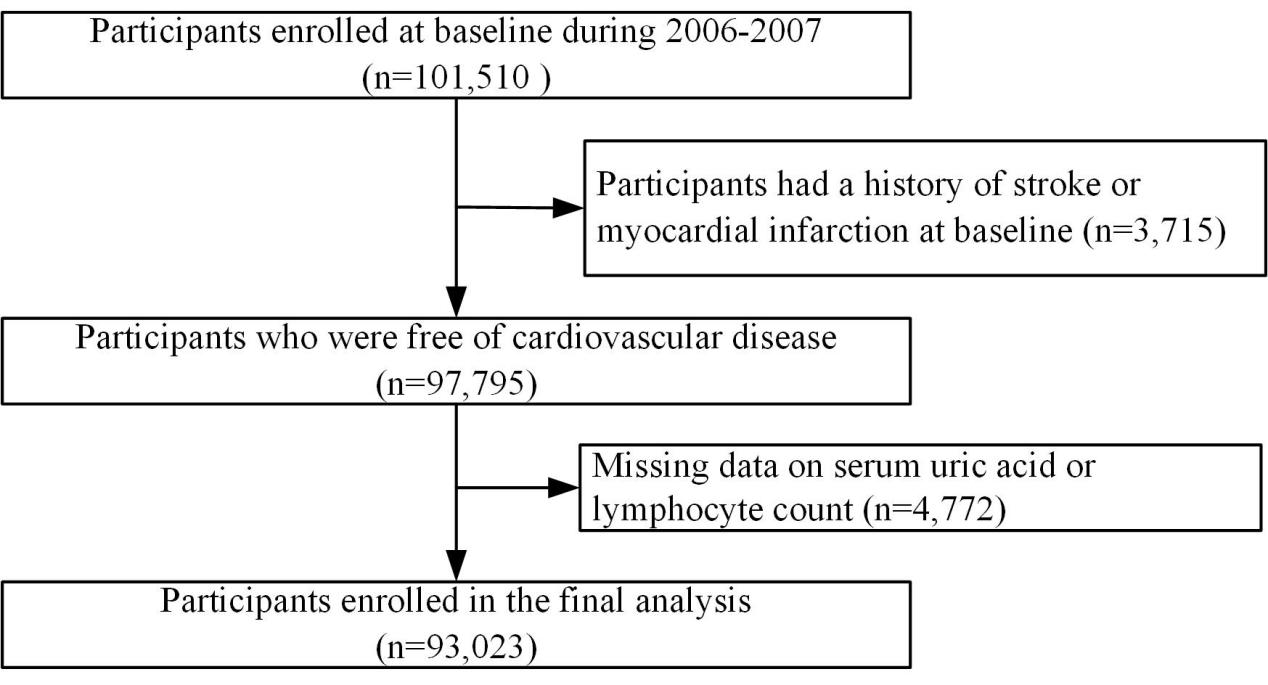


Figure S1. The flowchart of the study.


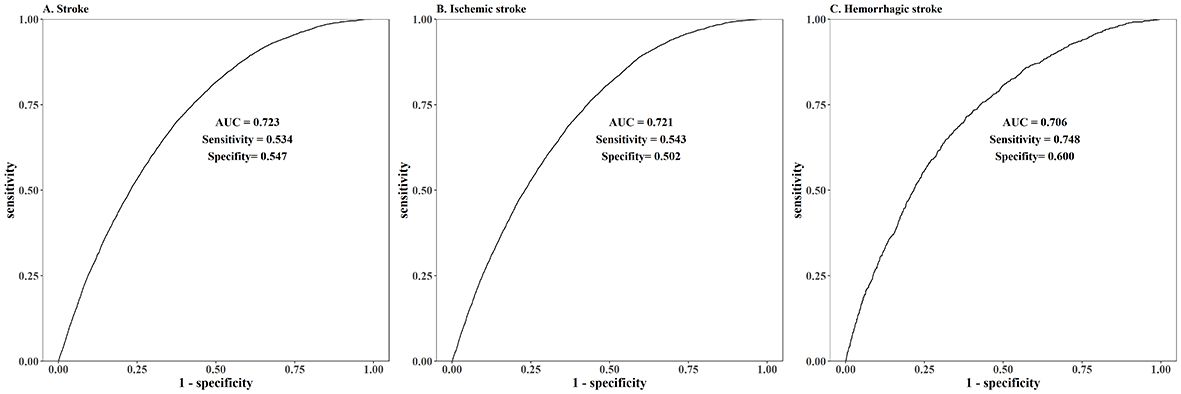


Figure S2. Receiver operative characteristics curve and cutoff value of uric acid to lymphocyte count ratio for incident stroke.

Abbreviation: AUC, area under curve


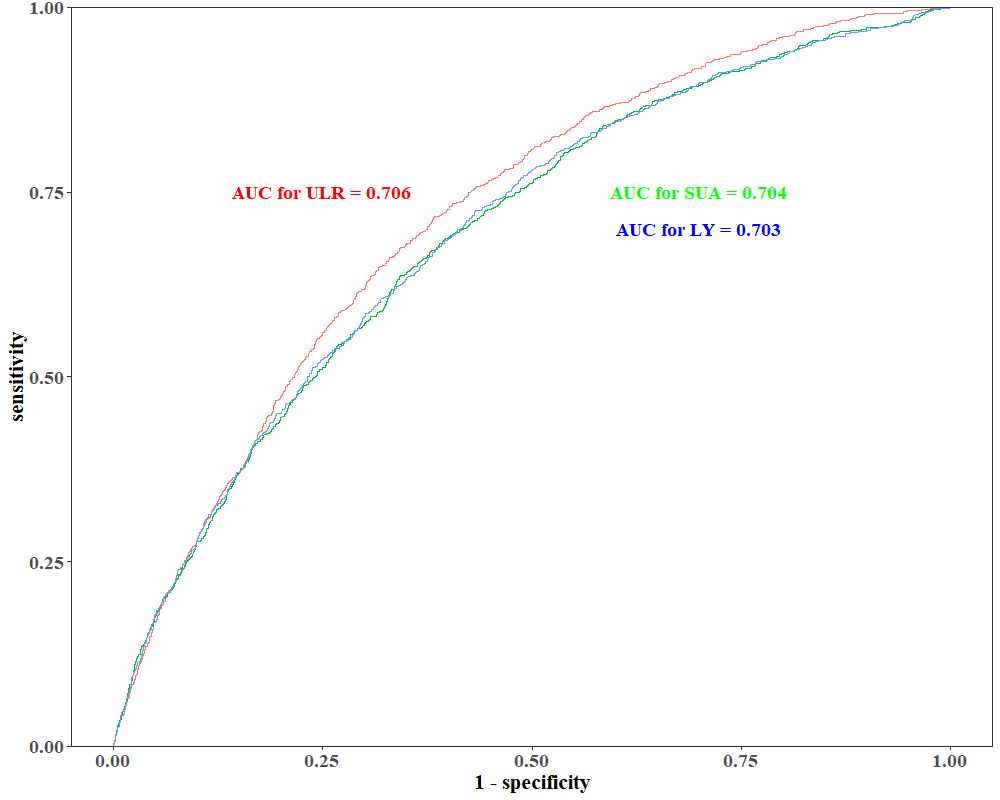


Figure R3. Receiver operative characteristics curve ULR, SUA, lymphocyte counts for incident hemorrhagic stroke.

Abbreviation: AUC, area under the curve; LY, lymphocyte counts; SUA, serum uric acid; ULR, uric acid to lymphocyte count ratio
